# Evaluation of the Disinfection Efficacy of Er-YAG Laser Light on Single-Species Candida Biofilms: Systematic Review

**DOI:** 10.3390/microorganisms13040942

**Published:** 2025-04-19

**Authors:** Diana Dembicka-Mączka, Magdalena Gryka-Deszczyńska, Jacek Sitkiewicz, Aleksander Makara, Jakub Fiegler-Rudol, Rafał Wiench

**Affiliations:** 1 Dental Office—Artistic Smile Studio, 61/1 Krakowska Street, 33-100 Tarnów, Poland; 2 Dentalove Clinic Ltd., 19 Borzymowska Street, 03-565 Warsaw, Poland; magdalena.grykaaa@gmail.com; 3 GoodLight Clinic, Victoria Bridge Road, Bath BA2 3GG, UK; jacek@goodlightclinic.co.uk; 4 Luxmed Dentistry, 20a Rejtana Street, 35-310 Rzeszów, Poland; alek.makara@gmail.com; 5 Department of Periodontal and Oral Mucosa Diseases, Faculty of Medical Sciences in Zabrze, Medical University of Silesia, 40-055 Katowice, Poland; s88998@sum.edu.pl (J.F.-R.); rwiench@sum.edu.pl (R.W.)

**Keywords:** resistance, chronic atrophic stomatitis, photothermal effect, optimal parameters, hyperplastic lesions

## Abstract

The relevance of the current study is to increase the resistance of fungal biofilms to traditional disinfection methods. The aim of the study was to determine how effectively Er:YAG laser light inhibits single-species Candida biofilms. The study involved a systematic review of 57 scientific publications (2015–2024) selected according to specific criteria, followed by an assessment of quantitative and qualitative indicators of colony-forming unit reduction. The results show that under optimal parameters (power 1.5–3.9 W and duration 60–90 s), the Er:YAG laser can reduce the number of viable *Candida albicans* cells by an average of 70–90%, and when combined with sodium hypochlorite or chlorhexidine solutions, this figure can exceed 90%. Separate in vitro tests show that *Candida glabrata* and *Candida tropicalis* require higher power or longer exposure to achieve a similar effect, while the use of the Er:YAG laser on titanium and dental surfaces minimizes damage to the substrate and effectively removes the biofilm matrix. In addition, laser treatment accelerates tissue regeneration and helps reduce the number of cases of reinfection, which is confirmed by the positive dynamics in clinical practice. Data analysis using confocal microscopy and microbiological seeding indicates a significant disruption of the biofilm structure and increased permeability to antimycotics after laser exposure. Er:YAG laser disinfection method is promising in counteracting fungal biofilms, especially for surfaces with a high risk of microbial colonization. The practical value lies in the possibility of developing standard protocols for the clinical use of the laser, which will increase the effectiveness of treatment and prevention of Candidal lesions.

## 1. Introduction

*Candida* biofilms pose a serious threat in clinical practice due to their ability to adhere to various surfaces and resist traditional disinfection methods (including chemical and heat treatments, antiseptic solutions, and mechanical cleaning) [1,2,3,4,5]. Their persistence is especially problematic in clinical contexts involving prosthetic devices, endodontic treatments, and immunocompromised patients, where biofilm-associated infections can lead to recurrent and difficult-to-treat conditions. These settings present significant obstacles due to the limited penetration of antifungal agents into biofilms and the high resistance of sessile cells compared to their planktonic counterparts [3,4,5]. In this regard, the issue of finding effective and at the same time gentle methods of surface treatment is of particular relevance. The Er:YAG laser is attracting attention as an innovative disinfection tool: it provides high precision and potentially minimizes tissue damage around the treated area. This approach can help reduce the risk of fungal recurrence and reduce the need for aggressive chemicals, making it a promising area in modern dentistry and general medical practice. As for the works of various researchers, the work of Deeb et al. [1] focused on the problem of effective destruction of microorganisms in dental practice. Scientists studied the effect of Er:YAG laser radiation in combination with antiseptic substances such as sodium hypochlorite (NaOCl), chlorhexidine (CHX), and hydrogen peroxide on bacterial viability. The results showed that this combination significantly reduces the number of microorganisms, making this approach promising for dental treatment. At the same time, the study did not investigate the long-term effects of the method on the oral microflora and its possible consequences for patient tissues. In the work of Reddy et al. [2], the authors focused on reducing the number of bacteria that cause root caries using laser radiation (Er:YAG and CO_2_) and antiseptic agents, including hydrogen peroxide, sodium NaOCl, CHX and sodium fluoride. Researchers have found that the combined use of laser and chemical agents has a stronger bactericidal effect than their separate use. However, the impact of this approach on healthy tooth tissue and its effectiveness in real clinical settings remained outside the scope of the study. The study by Datla et al. [3] was aimed at evaluating the effectiveness of the Er,Cr:YSGG laser method in removing biofilms from root canals in comparison with ultrasonic systems and traditional rinsing. The scientists conducted a laboratory study on 160 extracted molars that were artificially infected with *Candida albicans*, *Staphylococcus aureus*, *Streptococcus mutans* and *Enterococcus faecalis*. The researchers found that laser treatment had the highest level of microbial elimination compared to ultrasonic cleaning, while the least effective method was conventional syringe rinsing. An important finding was that in the apical region of the root canal, ultrasonic methods showed a higher residual amount of microbes, while the laser effectively cleaned even the most difficult areas. However, the study did not evaluate the long-term effects of such therapy and the possible side effects of laser irradiation on tooth tissue Zorlu et al. [4] conducted an experiment on 140 human premolars that were sterilized and infected with these pathogens. They found that laser treatment with Er:YAG-PIPS was as effective as NaOCl, while Er,Cr:YSGG laser showed a significantly lower level of antimicrobial activity. The combination of the Er:YAG-PIPS laser and NaOCl gave the highest level of microbial destruction. However, the study did not evaluate the effect of laser treatment on the structural integrity of dentin, which may be important for clinical use. The study by Valenti et al. [5] was aimed at assessing the effect of Er:YAG laser on the microbial flora of carious lesions containing *Candida* spp., *Streptococcus* spp. and *Lactobacillus* spp. The researchers investigated two methods of carious lesions treatment: traditional mechanical preparation and Er:YAG laser therapy. It was found that laser treatment significantly reduced the total number of microorganisms, including *Candida albicans*, and was less invasive than traditional methods. An important conclusion was that the Er:YAG laser may be particularly useful for treating caries in children or patients with hypersensitivity. However, the study did not evaluate the long-term effect of laser treatment on enamel remineralization. Wiench et al. [6] investigated the effectiveness of photodynamic therapy (aPDT) against *Candida albicans*, *C. glabrata* and *C. krusei* on acrylic surfaces. The use of a diode laser (635 nm) together with toluidine blue was effective in reducing colony-forming units (CFUs). However, the study did not take into account possible clinical factors such as saliva exposure or biofilm dynamics. Another study conducted by Tyczkowska-Sieroń et al. [7] studied the effect of cold plasma on the fungus *Candida albicans*, which is a common cause of fungal infections in humans. Scientists analyzed how the genetic and phenotypic characteristics of the fungus change after cold plasma treatment to assess its potential as an antimicrobial method. The study showed that plasma induces significant mutations that can alter the virulence of *Candida albicans*, opening up opportunities for new therapeutic strategies. However, the authors did not investigate the possible impact of plasma on human cells and the duration of the changes it causes in fungal cells. The work of Staniszewska [8] is devoted to the analysis of virulence factors of different *Candida* species. The author discusses the mechanisms that allow these fungi to adapt to the human environment, promote colonization and cause infectious diseases.

The study summarizes the molecular mechanisms of *Candida* pathogenicity and their role in the development of candidiasis, concluding that different strains have specific mechanisms of survival and resistance to the immune system. At the same time, the work is of a review nature and does not contain its own experimental data, which limits its use for the development of new therapeutic approaches. Thus, the aim of this study was to determine the effectiveness of the Er:YAG laser in the suppression of single-species *Candida* biofilms. The idea of this systematic review is to complement and encourage further research into the dental applications of this growing field [9,10,11,12,13,14,15].

## 2. Methods

**Figure 1:**
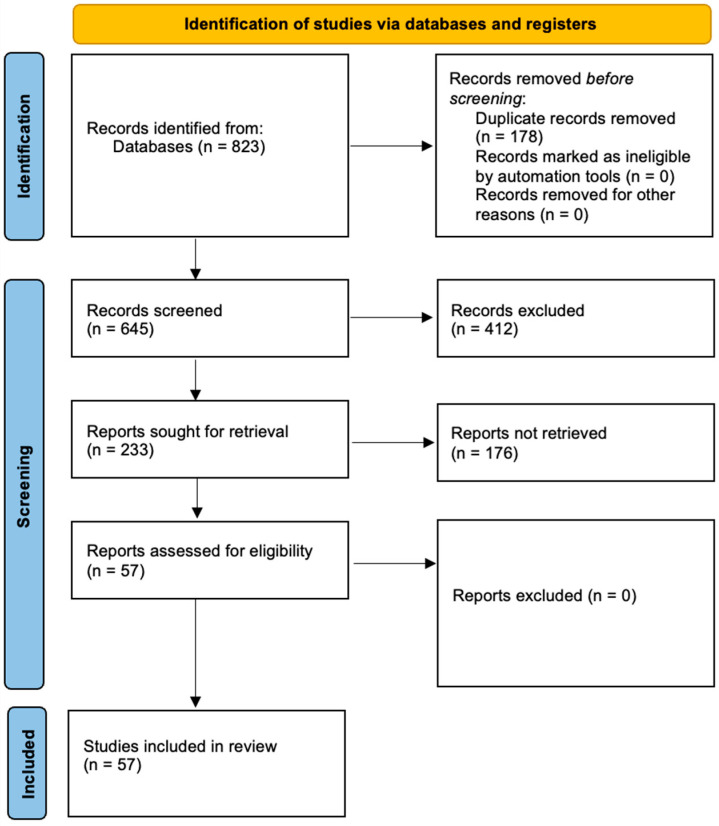
Flowchart of the process for selecting studies for a systematic review (PRISMA).

The current study was conducted in the format of a systematic review, which was carried out between January and December 2024. It was registered in PROSPERO with the number CRD420251011872. The analysis included publications published no earlier than 2015, covering the last 10 years in the field of Er:YAG laser disinfection of single-species Candida biofilms. Data were collected in accordance with the recommendations of Preferred Reporting Items for Systematic Reviews and Meta-Analyses (PRISMA 2020) [16]. The following PRISMA scheme was followed at the stages of literature search and selection, as shown in Figure 1.

The sources were searched in the electronic databases PubMed/Medline, Google Scholar and Cochrane Library using a combination of keywords (in various variations, using the logical operators AND/OR): ‘Er:YAG laser’, “Candida”, “*Candida albicans*”, “biofilm”, “disinfection”, “antifungal therapy”, etc. The search results were uploaded to a common database for further duplicate checking and relevance selection. However, it should be acknowledged that this language restriction may have introduced language bias, potentially excluding relevant studies published in other languages and thereby limiting the comprehensiveness of the review. Restrictions were imposed only on articles in Polish and English; other languages were not considered due to the impossibility of full data verification. The initial selection was carried out by two independent reviewers who assessed the compliance of studies with the inclusion criteria: (1) population—single-species *Candida* biofilms (with a focus on *C. albicans*, if available, also non-albicans forms), (2) intervention—disinfection with Er:YAG laser, (3) comparison—if other disinfection methods (ultrasound, chemical agents, other lasers) are available, (4) outcomes—effectiveness of biofilm inhibition, reduction of colony-forming units (CFU), improvement of clinical condition, (5) types of studies—original experimental or clinical (in vitro, in vivo), reviews and meta-analyses. The list excluded articles without clear quantitative indicators of laser exposure, reviews without detailed methodology, and publications without access to the full text. Two main sets of methods were used to write the systematic review. The first block includes methods for selecting and analyzing publications: formulating search queries, double-checking relevance, reconciling conflicting cases, and standardized extraction of key data (study type, sample characteristics, laser intervention parameters, main outcomes, risk of bias). This approach ensured consistency and reliability of the procedure, as well as transparency in accordance with the PRISMA 2020 guidelines. The second block includes methods for assessing the quality of the included studies: for clinical trials, the Cochrane Collaboration’s recommendations on the risk of bias (random sequence generation, allocation concealment, blinding, etc.) were applied, and for laboratory in vitro studies, a qualitative assessment was performed, considering the correctness of the experimental design, reproducibility, and the availability of control groups. This made it possible to determine the degree of reliability of the available conclusions and to take a balanced approach to their generalization. The approaches to interpreting the results were based on a comparison of quantitative and qualitative indicators of Er:YAG laser efficiency. The results were systematized according to disinfection methods, biofilm characteristics (thickness, hyphal form, resistance to traditional antimycotics) and type of study (in vitro, in vivo). If the characteristics of the papers differed significantly, narrative synthesis was used. In the presence of sufficiently homogeneous quantitative data, meta-analytical data pooling was potentially considered, but in most cases, descriptive comparisons were implemented due to the diversity of trial protocols.

## 3. Results and Discussion

### 3.1. Comparative Analysis of the Effectiveness of Er:YAG Laser Against Single-Species Candida Biofilms

The disinfectant effect of Er:YAG laser radiation against single-species *Candida albicans* biofilms depends on its physical parameters, including wavelength, pulse energy and penetration depth. The wavelength of the Er:YAG laser is 2940 nm, which ensures high absorption in water and biological tissues. This feature allows for the effective destruction of fungal biofilm structures, contributing to its elimination. One of the key parameters affecting the disinfection efficiency is the laser pulse energy. Studies show that increasing the exposure of an Er:YAG laser to 90 s significantly reduces the number of viable *Candida albicans* cells, which is explained by photothermal and photoacoustic effects. Laser radiation disrupts the fungus cell membranes and inhibits the growth of its colonies. The use of a combination of laser radiation and chemical agents, such as NaOCl or ethylenediaminetetraacetic acid (EDTA), further improves the efficiency of destroying the fungal biofilm [17]. The depth of penetration of the Er:YAG laser into biological tissues depends on its power and pulse mode parameters. In the case of *Candida albicans* biofilms formed on dental and titanium surfaces, laser radiation can penetrate to a considerable depth without damaging the underlying tissues. For example, when a laser is applied in a pulsed mode on titanium surfaces, it has been shown that the laser effectively destroys the fungal biofilm without adversely affecting the implant structure [18]. This is important in the context of dental implantology, as it reduces the risk of infectious complications. In addition, the use of a laser with a lateral spiral tip allows achieving high efficiency in the elimination of *Candida albicans* biofilms. Exposure to an Er:YAG laser in this configuration leads to a significant reduction in the number of colony-forming units (CFU), which confirms its high efficiency against fungal infections [19]. A wide range of methods, including microbiological, spectroscopic, and imaging technologies, are used to assess *Candida* viability in the study of the antifungal effect of the Er:YAG laser. One of the most common approaches is microbiological seeding, which allows for determining the number of colony-forming units (CFU) and assessing the reduction of viable cells after laser exposure. For example, the method of dilution and incubation on agarified media allows researchers to compare the effectiveness of laser disinfection by the number of surviving *Candida albicans* cells before and after laser irradiation [20]. Confocal laser scanning microscopy (CLSM) is another important method that allows studying the structure of *Candida* biofilms and analyzing the effectiveness of laser destruction of fungal structures. By using fluorescent dyes such as propidium iodide (PI), it is possible to differentiate between viable and non-viable cells in the biofilm. This method provides highly accurate visualization of the cell layer structure and changes in morphology after laser irradiation [21]. Biosensing methods are also used to assess the viability of *Candida*, especially in studies involving changes in cell wall composition or disruption of ergosterol synthesis. The use of laser desorption ionization mass spectrometry (MALDI-TOF MS) allows for the rapid detection of changes in cellular protein composition and metabolic pathways after laser irradiation [22]. In addition, cell membrane permeability tests using dyes such as propidium iodide allow assessing membrane damage and cell death caused by laser and its combinations with chemical agents [23]. Therefore, the most common methods for assessing *Candida* viability in studies of the disinfectant effect of Er:YAG laser are microbiological seeding, which allows for the assessment of colony survival, confocal laser microscopy, which provides detailed visualization of biofilms, and biosensing methods, which include spectroscopic and mass spectrometric approaches for analyzing cellular status. The combination of these methods allows for a comprehensive assessment of the laser’s effect on fungal cell viability and optimization of its application modes to improve antifungal efficacy. The effect of exposure time and Er:YAG laser power on the survival of *Candida albicans* in laboratory and clinical studies is a key factor in determining the optimal parameters of disinfection therapy. Studies have shown that increasing the irradiation time significantly reduces the viability of fungal cells, although the effect largely depends on the radiation intensity and additional treatments. Experimental studies have demonstrated that the Er:YAG laser with a standard wavelength of 2940 nm has a pronounced fungicidal effect, which increases with increasing exposure time. When comparing the modes of short (30 s) and prolonged (90 s) irradiation, it was found that the number of viable *Candida albicans* cells decreases significantly more in 90 s than in 30 s, which is confirmed by microbiological analysis of colony-forming units [24]. At the same time, laser treatment with a power of 2 W in a non-contact mode significantly reduces the number of colonies, but its effectiveness is lower compared to traditional antimycotics such as nystatin. One promising approach is to combine Er:YAG laser irradiation with antiseptic solutions. For example, the use of the laser in combination with 0.5% NaOCl or 0.03% CHX significantly improves fungal cell death in a shorter exposure time. The combined effect of the laser and disinfectant reduces the required exposure time, minimizing thermal damage to surrounding tissues [1]. It is also known that changing the laser power affects its effectiveness. Er:YAG laser radiation at the level of 1.8 J/cm^2^ and 3.9 J/cm^2^ provides an optimal reduction in fungal viability without significant heating of the surrounding tissues. However, when the energy density is increased to 5.8 J/cm^2^, an increased death of *Candida albicans* is observed without damaging the basic structure of the treated surface, which is critical in dental practice [18]. In general, laboratory studies confirm that the optimal Er:YAG laser exposure parameters for maximum antifungal effect depend on the combination of exposure time and power. The use of 3.9 J/cm^2^ for 60–90 s ensures effective elimination of fungal colonies, especially when combined with antiseptic solutions. Comparison of the effectiveness of the Er:YAG laser with traditional methods of controlling *Candida albicans*, such as chemical antiseptics and ultrasonic treatment, shows that laser therapy is a promising approach to destroying fungal biofilms, especially in dental and surgical practice. In vitro and in vivo studies demonstrate that exposure to laser radiation significantly reduces the viability of *Candida*, although its effectiveness depends on energy parameters and exposure time. The Er:YAG laser has a powerful antimicrobial effect due to photoacoustic and photothermal mechanisms of action. Studies have shown that laser irrigation in combination with chemical antiseptics such as NaOCl or chlorhexidine (CHX) significantly increases the disinfection efficiency compared to monotherapy with either method. For example, the combined use of an Er:YAG laser with 0.5% NaOCl demonstrated the greatest reduction in viable *Candida albicans* cells, while the use of a chemical agent or laser irradiation alone had a lower effect [2]. Comparison of the effectiveness of laser disinfection with ultrasonic treatment shows that the Er:YAG laser has an advantage in destroying *Candida* biofilms. It is known that *Candida* biofilms are much more resistant to traditional antiseptic agents (CHX, hydrogen peroxide, povidone-iodine), which makes them difficult to completely eliminate. In in vitro studies, the Er:YAG laser was able to significantly reduce the number of colony-forming units (CFUs), while ultrasonic irrigation did not produce similar results [19]. The Er:YAG laser has also demonstrated significant efficacy in dental therapy, especially in the treatment of oral candidiasis. In vivo studies confirm that the use of a 1.6 W laser for 20 s reduces the number of *Candida* colony-forming units by almost 90%, which is comparable to or even better than the effectiveness of traditional antiseptic solutions (CHX 0.12%, hydrogen peroxide 3%, povidone-iodine 10%) [20]. In general, a comparative analysis of in vitro and in vivo studies confirms that the Er:YAG laser is a highly effective technology for controlling the growth of *Candida albicans*. It can be used as a monotherapy or in combination with chemical agents, providing effective destruction of fungal biofilms and minimizing the risk of infection recurrence. Compared to traditional methods, laser therapy demonstrates greater efficacy and safety, especially in cases where it is necessary to avoid chemical exposure to tissues or reduce mechanical impact on the treated surface. The Er:YAG laser interacts with *Candida albicans* biofilm exopolysaccharides, in particular glucans, causing structural changes that increase the effectiveness of antifungal therapy. The main mechanism of its action is the destruction of the biofilm polymer matrix due to the photothermal effect, which weakens intermolecular bonds in the structure of exopolysaccharides. This is confirmed by studies that show a decrease in the thickness of *Candida* biofilms after laser irradiation, as well as an increase in the permeability of antimycotic agents due to the weakening of the glucan barrier [25,26,27,28]. During the experimental tests, laser treatment led to a significant reduction in the density of *Candida* biofilm, as confirmed by scanning electron microscopy and confocal microscopy. The high energy parameters of the Er:YAG laser (2940 nm, 1.6 W, 40 Hz) cause structural changes in exopolysaccharides, which makes the biofilm more fragile and susceptible to antimicrobial therapy [17]. In addition to destroying the polymer matrix, laser radiation reduces the adhesion of *Candida* cells to surfaces, which makes it difficult to re-form the biofilm. This is due to the effect on the protein components of the cell wall, which are responsible for the adhesion of fungi to tissues. The combined use of the Er:YAG laser and antimycotic drugs (e.g., fluconazole or amphotericin B) has been shown to increase the effectiveness of therapy by facilitating the penetration of drugs into the deeper layers of infected tissue [29]. Thus, the Er:YAG laser effectively destroys the glucan matrix of *Candida* biofilms, which leads to a violation of their structural integrity and increases the permeability of antimycotic agents. This opens up the prospects for combined laser therapy to improve the treatment of fungal infections, especially in cases where *Candida* biofilms are resistant to conventional treatments. It is worth noting that the Er:YAG laser is a highly effective tool for disinfecting *Candida* biofilms due to the combination of photothermal and photoacoustic effects. The combination of laser exposure with chemical antimicrobial agents enhances the antifungal effect and accelerates the destruction of biofilms, and careful selection of parameters (power, exposure time) minimizes thermal damage to tissues. To improve the efficiency, it is worth optimizing the parameters (power and exposure time) of the Er:YAG laser according to the density of the *Candida* biofilm. Use combined approaches (laser + antiseptics) to enhance the antifungal effect. Monitor the condition of tissues after irradiation and adjust the exposure regimen if necessary, especially in patients with comorbidities. The rising challenge of antifungal resistance necessitates exploring multifaceted treatment approaches. Hetta et al. (2025) discuss novel therapeutic pathways beyond conventional antifungals, including nanotechnology, drug repurposing, and immunotherapy, as promising avenues to combat resistant fungal infections [30]. These strategies aim to enhance treatment efficacy and overcome the limitations of existing antifungal agents [30]. Natural compounds have also shown potential in antifungal therapy. Pinna et al. (2025) evaluated an ophthalmic spray containing Biosecur^®^ citrus extract (Oftasecur^®^) and found it effective against *Candida auris* and *Candida albicans*, including their biofilms on contact lenses [31]. Similarly, Donadu et al. (2021) demonstrated that essential oil from Ruta graveolens exhibits antifungal activity against various *Candida* species, including strains resistant to conventional antifungals. These findings suggest that integrating natural compounds with laser therapy could enhance antifungal efficacy [32]. Combining Er:YAG laser treatment with these complementary approaches may offer a synergistic effect, improving outcomes in managing *Candida* infections. Further research into such integrative therapies could lead to more effective and comprehensive antifungal treatment protocols.

### 3.2. Comparison of Er:YAG Laser and Er,Cr:YSGG Laser, Its Antifungal Capabilities in Dentistry

The Er:Cr:YSGG laser and the Er:YAG laser differ in wavelength, operating modes and safety level in the treatment of oral candidiasis. Er:YAG laser emits at a wavelength of 2940 nm, while Er,Cr:YSGG has a wavelength of 2780 nm, which causes a different degree of absorption in water and hydroxyapatite, and therefore differences in the mechanisms of action on tissues. Er:YAG provides a predominantly photoabsorptive effect, which allows for precise vaporization of the affected tissue without significant heating of the surrounding structures. Er,Cr:YSGG, due to its wavelength and the combination of water and air in the energy delivery system, provides a softer effect, which helps to reduce thermal damage to tissues and allows treatment of deeper layers of the mucous membrane [33]. In terms of safety, studies show that the Er,Cr:YSGG laser creates a lower level of thermal damage compared to Er:YAG with the same energy parameters. Erbium laser with a wavelength of 2780 nm allows using lower power without losing efficiency, which is especially important when treating the oral mucosa. In addition, the analysis of postoperative healing of patients showed that the use of Er,Cr:YSGG was accompanied by less postoperative sensitivity and discomfort compared to Er:YAG [34]. Both lasers have demonstrated efficacy in the treatment of candidiasis, but their mechanism of action has certain differences. Er:YAG laser provides more pronounced destruction of the biofilm and a high antifungal effect, while Er,Cr:YSGG is characterized by less trauma to the tissues and a more comfortable postoperative period, making Er,Cr:YSGG the best choice in cases where it is important to minimize pain and inflammation, and Er:YAG is more effective when radical removal of infected tissue and deep sanitation of the affected area is required. The Er,Cr:YSGG laser demonstrates high efficiency in disinfection of dental prostheses and implants, on which *Candida albicans* biofilms are formed. The main mechanism of laser action is its ability to destroy the polymeric matrix of the biofilm through photothermal and photoacoustic effects, which reduces the adhesion of fungal cells to surfaces. Studies show that the Er,Cr:YSGG laser can significantly reduce the number of *Candida* colony-forming units (CFUs) on titanium implants and also reduce the thickness of biofilms [35]. The Er,Cr:YSGG laser provides effective sterilization of dental implant surfaces, reducing the formation of biofilms without damaging their structural integrity. In vitro studies have confirmed that laser radiation significantly improves the antifungal effectiveness of titanium surfaces, reducing the number of viable *Candida* cells on implants, especially when combined with antimicrobial coatings [36]. In the treatment of dental prostheses, the Er,Cr:YSGG laser has proven to be an effective tool in the fight against fungal colonies, ensuring the removal of biofilms without damaging the prosthesis material. Studies have shown that laser treatment significantly reduces the microbial load on the surface of dentures, preventing further colonization by *Candida albicans* [37]. In general, Er,Cr:YSGG laser is a promising method for disinfection of dental materials, providing effective destruction of *Candida albicans* biofilms and reducing the risk of recolonization without significantly affecting the physical properties of implants and prostheses. The Er,Cr:YSGG laser has shown significant efficacy in reducing the risk of reinfection of *Candida albicans* in patients with recurrent forms of denture-related stomatitis. Due to its wavelength of 2780 nm, this laser has a pronounced photothermal effect, which allows not only to eliminate *Candida* biofilms, but also to change the surface properties of dental materials, reducing the adhesion of fungal cells. Studies have shown that laser disinfection of implants and dentures leads to a significant reduction in *Candida* colony-forming units, especially when used repeatedly at regular intervals [38]. Clinical studies show that the use of the Er,Cr:YSGG laser in combination with mechanical treatment and antimicrobial solutions provides a longer-lasting antifungal effect compared to chemical disinfection. Repeated treatments are recommended every 3–6 months for patients at high risk of recurrence, as a gradual increase in fungal colonization is observed after this period [39]. Studies have also shown that the Er,Cr:YSGG laser helps to reduce inflammation in patients with *Candida albicans* stomatitis by reducing biofilm activity and improving tissue healing. In addition to reducing the number of fungal colonies, laser treatment helps to reduce pain and gum inflammation in patients, which is especially important for people with prolonged use of dentures [40]. A more detailed comparison of Er:YAG laser parameters with Er,Cr:YSGG is presented in Table 1.

The Er,Cr:YSGG laser shows significant potential for use in patients with systemic risk factors, such as diabetes and immunodeficiency, which increase the likelihood of Candidal lesions. Its benefits include the effective destruction of *Candida albicans* biofilms, improved tissue healing and reduced inflammation. Laser radiation at a wavelength of 2780 nm has a pronounced antimicrobial effect due to photoacoustic and photothermal effects, which reduces the risk of recurrence of fungal infection without the need for systemic antimycotics [42]. Patients with diabetes often have impaired tissue regeneration and increased susceptibility to infections, which complicates the treatment of dental lesions. The use of Er,Cr:YSGG laser improves blood supply to the affected areas, stimulates cell proliferation and accelerates healing, which is especially important for diabetic patients with Candidal stomatitis or peri-implantitis [43]. In patients with immunodeficiency, the Er,Cr:YSGG laser can be an effective method of controlling *Candida albicans* by reducing the microbial load without the need for long-term antimicrobial use. Laser treatment can minimize the use of chemical antiseptics, which is important for patients with hypersensitivity to drugs or a weakened immune response [44]. However, there are certain limitations. In patients with impaired thermoregulation, which is typical for diabetics, laser therapy can increase the risk of excessive tissue heating, which requires careful monitoring of exposure parameters. In addition, in cases of deep infectious processes, laser treatment may need to be supplemented with traditional antifungal methods to achieve the optimal therapeutic effect. Thus, the Er,Cr:YSGG laser is a promising tool for the treatment of Candidal lesions in patients with systemic risk factors, providing effective disinfection, stimulating healing and reducing the need for pharmacological treatment. However, its use requires an individual approach, especially in patients with comorbidities that may affect thermoregulation and immune response. The Er,Cr:YSGG laser is more delicate for tissues and provides a more comfortable postoperative period, while the Er:YAG laser is more effective in cases of deep rehabilitation of the affected area. Both lasers are suitable for the treatment of *Candida*-associated lesions, in particular when used on implants and prostheses, and the choice of a specific technology depends on the clinical situation and individual patient characteristics. Thus, the Er:YAG laser should be chosen for radical removal of fungal biofilms and deeper tissue rehabilitation, and Er,Cr:YSGG should be used in cases where it is important to reduce postoperative discomfort. For implants and prostheses, use laser treatment with repeated sessions every 3–6 months to minimize the risk of reinfection. Particular attention should be paid to patients with diabetes and immunodeficiency, carefully controlling the laser parameters.

### 3.3. The Effect of Laser on Candida albicans and Non-abicans Forms

Laboratory studies show that *Candida albicans* and non-albicans species (*Candida glabrata*, *Candida tropicalis*, etc.) have different sensitivities to the Er:YAG laser, which is due to differences in cell wall structure and fungal cell metabolism. *C. glabrata* and *C. tropicalis* show higher resistance to Er:YAG laser, which is associated with an increased amount of β-glucans in the cell wall and the ability to quickly adapt to environmental changes [45]. Studies have shown that to achieve a similar fungicidal effect on *C. glabrata* and *C. tropicalis*, a higher Er:YAG laser power or longer irradiation time may be required. This confirms the need for an individual approach to the treatment of different *Candida* species, especially in cases of Candidal lesions in patients with a weakened immune system [46]. Thus, *C. albicans* demonstrates the highest sensitivity to the Er:YAG laser among the studied species, while *C. glabrata* and *C. tropicalis* require more intense exposure or a combination of laser therapy with antimicrobial agents to achieve effective elimination of fungal cells. The Er:YAG laser is a promising treatment for *Candida*-associated oral disorders such as black hairy tongue and chronic atrophic stomatitis. Due to its wavelength of 2940 nm, this laser is effectively absorbed by water, which allows for precise treatment of the affected tissue without significant heating of the surrounding structures. Black hairy tongue is characterized by excessive keratinization of the tongue’s filiform papillae, which contributes to the accumulation of food debris, bacteria and fungi, including *Candida albicans.* One of the main methods of treatment is the mechanical or chemical removal of excess keratinous formations, but Er:YAG laser irradiation allows for more delicate and effective cleaning of the tongue surface. Studies have shown that after laser application, there is a significant reduction in the thickness of hyperkeratosis, elimination of pigmented areas and restoration of normal tongue color, as well as improvement of taste sensations in patients [47]. Laser exposure also helps to reduce the number of *Candida* colonies and stimulates epithelial regeneration, making this method effective even in cases of recurrent candidiasis. The use of the Er:YAG laser in patients with *Candida*-associated lesions provides significant advantages over conventional treatments. Its ability to remove infected tissue, destroy fungal biofilms, and improve mucosal regeneration allows for faster clinical improvement and reduces the likelihood of recurrence. This method is especially effective in combination with topical antiseptic solutions or antifungal drugs, which allow for maximum therapeutic effect [48]. Studies also show that the procedure is safe, has no serious side effects, and can be used in patients with concomitant systemic diseases. Er:YAG laser therapy demonstrates significant potential as an effective and minimally invasive method of treating *Candida*-associated lesions, which may become a new standard in the comprehensive approach to the treatment of oral candidiasis. Laser treatment causes significant changes in the phenotype and genotype of *Candida* non-albicans, in particular *Candida glabrata* and *Candida tropicalis*, which is reflected in changes in cell morphology, colony size and expression of genes associated with stress resistance. In vitro studies show that laser irradiation causes a decrease in colony size and changes in the structure of the cell wall, which leads to a violation of its integrity and reduced adhesion. This is particularly pronounced in *C. glabrata*, which has a denser cell wall and lower ergosterol content, which may make it more resistant to laser exposure. After laser treatment, *C. tropicalis* shows a change in cell shape from the typical oval to a more elongated one, which may indicate that the fungus is trying to adapt to a new stressful environment [49,50]. At the genotype level, studies have revealed increased expression of genes responsible for the stress response and survival mechanisms of fungal cells. In particular, in *C. glabrata* and *C. tropicalis*, after laser irradiation, there is an increase in the transcription of genes encoding heat shock proteins (HSP90, HSP70) and antioxidant enzymes (SOD2, CAT1), which helps cells to compensate for the damage caused by laser energy. This confirms the hypothesis of an adaptive mechanism of *Candida* survival under adverse conditions [7]. In vitro studies also demonstrate that certain species of *Candida* non-albicans, in particular *C. krusei*, can exhibit significantly lower sensitivity to laser exposure, which is manifested in the preservation of the structural integrity of colonies and less pronounced changes in cell morphology. This suggests that different mechanisms of response to laser treatment may be activated in different *Candida* species. In the case of *C. albicans*, a significant decrease in the number of viable cells was observed after laser exposure, while *C. tropicalis* and *C. glabrata* had more variable results depending on the laser irradiation parameters [24]. Thus, laser therapy causes profound changes in the phenotype and genotype of *Candida* non-albicans, which is manifested in changes in morphology, colony size, structural integrity of the cell wall, and activation of stress response mechanisms. Different species of *Candida* show different degrees of adaptation to laser exposure, which indicates the need to optimize the parameters of laser therapy for maximum effect against fungal infections. Laser disinfection has a significant impact on the clinical course of chronic *Candida*-associated lesions, such as resistant Candidal glossitis and hyperplastic lesions of the oral mucosa. Studies show that laser therapy can be an effective alternative or complement to traditional antimycotic treatment, especially in cases where fungal colonies are resistant to standard drugs. The Er:YAG laser is one of the most promising tools for the treatment of *Candida*-associated lesions, as its radiation effectively removes biofilms, destroys hyperkeratotic layers of the mucous membrane and creates favorable conditions for tissue healing. Clinical studies have shown that laser disinfection helps to reduce the number of colony-forming units of *Candida albicans* and *Candida* non-albicans, especially when combined with antimicrobial agents [49]. In cases of resistant Candidal glossitis, laser therapy not only reduces the severity of hyperplasia but also improves the general condition of the tongue mucosa, reducing inflammation and pain. Chronic hyperplastic Candidal leukoplakia is a pre-tumour condition that requires constant monitoring and effective treatment. Studies have shown that laser treatment can promote the regression of lesions, prevent the progression of pathology and reduce the risk of malignant cell transformation. The use of a CO_2_ laser or Er:YAG laser can effectively eliminate hyperkeratotic areas of the mucous membrane and create conditions for their regeneration, which is confirmed by positive clinical results [50]. Thus, the results of studies show that laser therapy is an effective method for controlling chronic *Candida*-associated conditions in the oral cavity. It not only reduces the fungal load, but also promotes the regeneration of affected tissues, reduces the risk of relapse and complications, and provides better control over pathological processes. Laser treatment of *Candida*-associated biofilms requires the adaptation of parameters such as power and exposure time, depending on the type of fungus. Studies have shown that *Candida albicans* form thicker biofilms consisting of deeper layers of exopolysaccharide matrix and a more branched hyphal structure, which makes them more sensitive to Er:YAG laser at moderate exposure parameters. At the same time, *Candida* non-albicans species, in particular *Candida glabrata* and *Candida parapsilosis*, show a denser matrix and less tendency to hyphal growth, which requires longer irradiation or increased laser power for effective biofilm destruction [51]. The physicochemical properties of biofilms affect their sensitivity to laser irradiation. *C. albicans* biofilms contain more ergosterol, which increases their vulnerability to the thermal effects of the Er:YAG laser, while *C. glabrata* demonstrates adaptation through increased expression of genes related to stress resistance and structural modification of the cell wall [52]. It has been established that using an Er:YAG laser with a power of 1.5–2.5 W for 30 s achieves effective destruction of *C. albicans* biofilms, while removal of *C. glabrata* and *C. tropicalis* biofilms may require an increase in power to 3.0 W or a longer exposure time (60–90 s). Experimental data confirm that *Candida* non-albicans biofilms show increased resistance to laser exposure due to the denser matrix and less water in their composition, which reduces the effectiveness of the photothermal effect of the laser. To compensate for this effect, it is recommended to use a pulsed Er:YAG laser mode with an increased frequency (40–50 Hz), which ensures more uniform energy penetration into the deeper layers of the biofilm [53]. Thus, for the effective treatment of *Candida albicans* and *Candida* non-albicans biofilms, laser therapy should be individually adapted. *C. albicans* biofilms can be destroyed with moderate Er:YAG laser parameters, while for more resistant *C. glabrata* and *C. tropicalis*, it is necessary to increase the power and duration of irradiation. A detailed analysis of the potential of the Er:YAG laser for various diseases of the oral cavity is given in Table 2.

Studies confirm that *Candida* can develop adaptations to repeated laser irradiation, including increased heat resistance, changes in membrane components and enhanced stress response mechanisms. A study of *C. tropicalis* showed that after prolonged exposure to high temperatures, this fungus can activate genetic adaptation mechanisms, including increased expression of heat shock proteins (HSP90, HSP70), antioxidant enzymes (SOD2, CAT1) and changes in membrane lipid composition to improve cell protection [56], indicating a potential risk of reducing the effectiveness of laser treatment in the case of repeated exposure. Other studies have shown that photodynamic therapy using laser irradiation can cause partial resistance in *C. albicans* if the fungus is not completely eradicated during the initial exposure. In vitro tests on *C. albicans* samples indicate that the fungus can change the structure of the cell wall, increasing the content of ergosterol, which reduces the effectiveness of further laser irradiation [57]. Studies also show that *C. albicans* can develop adaptive changes in metabolism that increase its resistance to laser exposure. For example, it has been found that after repeated laser irradiation, fungal cells show increased expression of membrane proteins and enzymes involved in maintaining cellular homeostasis under stress [58]. This indicates the need for careful control of laser treatment parameters and the development of combined therapeutic strategies to prevent fungal cell adaptation. Thus, there is evidence of the possible development of *Candida* adaptation to repeated laser irradiation through mechanisms of heat resistance, changes in membrane components, and metabolic adaptation. This can affect the long-term effectiveness of treatment, requiring the development of individual laser therapy protocols and combined strategies to maximize the eradication of fungal colonies. It is worth noting that the level of sensitivity of different *Candida* species to laser exposure depends on the characteristics of the cell wall and the ability to adapt. *C. albicans* is generally more vulnerable to the Er:YAG laser, while non-albicans species may require higher parameters and combined approaches. Despite the risk of adaptation, laser therapy remains an effective method, especially in cases of resistant candidiasis. For better implementation of the Er:YAG laser, the species of *Candida* should be determined before starting laser therapy. For *C. glabrata*, *C. tropicalis* and other non-albicans species, it is necessary to use higher parameters (higher power and longer exposure) or combine the laser with antimycotics. It is worth watching for possible signs of fungal adaptation during repeated sessions and changing the treatment protocol if necessary. A summary of the evidence is shown in Table 3.

### 3.4. Clinical Prospects for the Use of Er:YAG and Er,Cr:YSGG in Candidiasis

The Er:YAG laser is widely used in the treatment of *Candida*-associated oral lesions, including chronic atrophic stomatitis, acute pseudomembranous candidiasis and hyperplastic mucosal lesions. Its ability to selectively ablate tissues, destroy microbial biofilms and stimulate regeneration makes it effective in cases of resistant candidiasis that are not amenable to standard antimycotic drugs. Chronic atrophic stomatitis, especially in patients wearing removable dentures, is one of the main areas of application for the Er:YAG laser. Studies have shown that laser therapy reduces inflammation, removes fungal biofilms and stimulates epithelial regeneration. In vivo trials have shown a significant reduction in the number of *Candida albicans* colony-forming units after laser treatment, which allows for faster remission of chronic candidiasis [59]. Acute pseudomembranous candidiasis is another clinical indication for laser therapy, especially in patients with immunosuppression. Er:YAG laser irradiation can effectively remove pseudomembranes and fungal colonies, minimizing mucosal trauma. This is especially relevant in patients receiving radiotherapy or chemotherapy, as traditional antimycotic drugs may have limited efficacy or cause unwanted side effects [58,59]. Hyperplastic lesions of the oral mucosa, such as chronic hyperplastic candidiasis or leukoplakia with fungal complications, are also an indication of the use of the Er:YAG laser. Laser therapy helps to eliminate pathologically altered tissues, reduce the fungal load and prevent the risk of malignant cell transformation. Thus, the main clinical indications for the use of the Er:YAG laser are chronic atrophic stomatitis, acute pseudomembranous candidiasis and hyperplastic lesions of the oral mucosa. Laser treatment provides a rapid reduction of fungal colonization, stimulates tissue regeneration and reduces the risk of relapse, making it an effective method in the treatment of complex cases of oral candidiasis. Laser treatment of the oral cavity affects not only *Candida*, but also the overall microbial composition, including commensal bacteria and other fungi. Studies show that laser radiation can cause both a decrease in pathogenic microorganisms and potentially changes in the balance of normal microflora. The use of laser in combination with antimicrobial drugs has the potential to more selectively target pathogenic microflora without significantly damaging beneficial bacteria. Laser-based photodynamic therapy in combination with methylene blue or toluidine blue has been shown to be effective against *Candida glabrata*, *Candida krusei*, and *Staphylococcus aureus*, while leaving most of the commensal microflora unaffected [27,60]. In addition to the direct destruction of microbial cells, laser treatment can also affect the formation of biofilms. Studies have shown that laser therapy reduces the ability of bacteria to form biofilms, especially in the case of *Candida* spp. and *Streptococcus* spp. which can facilitate further treatment with antibacterial or antifungal drugs [60]. Thus, laser therapy has a significant effect on the microbial composition of the oral cavity, providing effective destruction of pathogens, in particular *Candida* and *Streptococcus* mutans. At the same time, a possible decrease in the number of beneficial bacteria requires a cautious approach to treatment and control of the microflora, especially with repeated use of laser techniques. Combined therapeutic approaches, such as photodynamic therapy, can contribute to a more selective effect, minimizing disruption of the normal oral microbiome. Laser therapy for *Candida* infections, especially in combination with antimycotics, can be accompanied by side effects, complications and certain limitations that affect its clinical use. It is known that laser therapy can cause local irritation of the mucous membrane, thermal damage to tissues, as well as changes in the microbial composition of the oral cavity, which can affect the balance of normal microflora. In studies using photodynamic laser therapy, it was found that prolonged or repeated irradiation can promote the adaptation of *Candida albicans* and *Candida auris*, which can complicate further treatment and increase the risk of recurrence [61]. A crucial aspect is the interaction of laser with antimycotics. Studies have shown that combining laser treatment with azole drugs can increase their effectiveness by increasing the permeability of the *Candida* cell wall. However, in some cases, a decrease in the sensitivity of fungi to antimycotics was observed after repeated exposure to laser irradiation, which may be due to the activation of stress resistance mechanisms [62]. In addition, combined laser and antimycotic therapy may increase the risk of epithelial tissue damage, especially in patients with comorbidities such as diabetes or immunodeficiency. Studies indicate possible inflammatory reactions, including increased redness, swelling, and tenderness at the laser sites, which may impair patient comfort and require additional therapy adjustments [63]. In general, laser therapy for *Candida* infections is a promising treatment method, but it requires careful monitoring of exposure parameters, individualized treatment regimens, and assessment of possible complications, especially in cases of combined use with antimycotic drugs. Laser treatment of oral candidiasis has a positive effect on the quality of life of patients, reducing pain, improving oral functionality and helping to restore the aesthetic characteristics of the mucous membrane. Studies have shown that laser-based photodynamic therapy is effective in eliminating the symptoms of chronic candidiasis, including pain, burning sensation and difficulty swallowing, which is especially important for patients undergoing cancer treatment or those with immunodeficiency conditions. The use of laser treatment in combination with methylene blue in patients with Candidal stomatitis led to a significant improvement in quality of life, pain relief, and normalization of swallowing and chewing function over an eight-week treatment period [64]. Laser treatment also promotes faster mucosal healing and minimizes the risk of recurrence, which is especially important for patients with chronic hyperplastic candidiasis. Studies evaluating the combined use of a semiconductor laser and 5-ALA photodynamic therapy have shown a significant reduction in inflammation and improvement in the overall condition of the mucosa. Patients reported a significant improvement in the aesthetic characteristics of the oral cavity and a reduced risk of infection recurrence without the need for additional surgical interventions [65]. In addition, laser therapy has a positive effect on patients with candidiasis resulting from radiotherapy. Patients with cancer who received laser treatment experienced an improvement in quality of life, a decrease in the severity of pain, and a restoration of the ability to eat normally without discomfort. The UW-QOL assessment confirmed that patients who underwent laser therapy had significantly better comfort, chewing, and overall functional status compared to those who received conventional treatment alone [66]. In general, laser therapy of oral candidiasis contributes to a significant improvement in the quality of life of patients by reducing pain, normalizing chewing and swallowing functions, and improving the aesthetic characteristics of the mucous membrane. The use of laser treatment as a standalone method or in combination with photodynamic therapy provides high efficiency in the treatment of chronic and recurrent forms of candidiasis, making it an important component of modern dental therapy. The prospects for the implementation of standard protocols for the use of Er:YAG lasers in dental clinics for the prevention and treatment of fungal biofilms are promising, given their effectiveness in removing biofilms and minimal invasiveness. Studies confirm that Er:YAG lasers show significant potential in dental treatment, providing antibacterial and antifungal effects due to their ability to ablate surface microorganisms without damaging underlying tissues. It has been established that laser treatment can be an effective alternative or complement to traditional methods of disinfecting dental surfaces, implants and oral mucosa [17]. One of the key advantages of the Er:YAG laser is its ability to effectively remove biofilms containing *Candida* and other pathogens that are resistant to standard antiseptics. In vitro studies have confirmed that the use of a laser in combination with endodontic irrigation provides more effective elimination of microorganisms compared to chemical disinfection methods [17]. Another promising area is the use of Er:YAG laser for the prevention of fungal infections in patients with dentures or implants. It is known that such patients are prone to the formation of persistent *Candida albicans* biofilms that can cause chronic stomatitis. Implementation of standardized protocols for laser disinfection of dentures can significantly reduce the risk of fungal infections and improve long-term prosthetic outcomes [67,68]. In addition, the implementation of standard protocols for the use of Er:YAG lasers requires further research to optimize energy and exposure time parameters for different clinical situations. Recent studies have proposed a laser technique for the treatment of peri-implantitis that can be adapted to combat fungal biofilms. This technique involves complete laser cleaning of the implant surface, which effectively destroys fungal cells and reduces the risk of inflammation [48]. Thus, the prospects for implementing standard Er:YAG laser therapy protocols in dental clinics are quite high. They include effective disinfection of the oral cavity, prevention of fungal biofilms on implants and prostheses, and expansion of the possibilities of laser therapy for the treatment of peri-implantitis and other dental complications. Further research is needed to create detailed clinical guidelines that would optimize laser exposure parameters and ensure safe and effective treatment. In summary, Er:YAG and Er,Cr:YSGG lasers open up new opportunities for the treatment and prevention of candidiasis in dentistry [69,70,71,72,73,74]. Their advantage lies in their high efficiency against fungal biofilms, minimal tissue damage and improved quality of life for patients. It is important to develop clear protocols for the use of laser therapy, considering the type of fungus, the patient’s condition and possible side effects. It is advisable to include Er:YAG or Er,Cr:YSGG laser in the complex treatment of chronic and recurrent forms of oral candidiasis, especially in the presence of resistance to traditional antimycotics. Regularly carry out laser disinfection of dentures and implants to prevent the formation of fungal biofilms. In patients with immunodeficiency or after radiotherapy, use laser methods with careful monitoring to minimize side effects and ensure rapid mucosal healing [68,69,70,71,72,73,74,75]. A comparison of Er:YAG to Er,Cr:YSGG is shown in Table 4.

## 4. Conclusions

Given the relevance of the problem of *Candida* resistance to traditional disinfection methods, the current study was conducted to comprehensively systematize the effect of the Er:YAG laser on single-species fungal biofilms. The data obtained confirm that laser energy at a wavelength of 2940 nm effectively destroys the structure of *Candida albicans* biofilms due to strong absorption in aqueous medium and photoacoustic effects. A detailed comparative analysis of in vitro and in vivo studies shows a significant reduction in colony-forming units when Er:YAG laser irradiation is combined with disinfectant solutions, and also indicates the importance of the optimal duration (60–90 s) and radiation power. A significant new aspect was the high efficiency of exopolysaccharide matrix destruction, which makes fungal cells more vulnerable to antimycotics; at the same time, *Candida* non-albicans forms show greater resistance and require enhanced parameters or combined strategies. A comparative review of Er:YAG and Er,Cr:YSGG revealed the advantages of the former in destroying denser fungal layers, while Er,Cr:YSGG is less traumatic for the surrounding tissues. The benefits of laser treatment for chronic *Candida*-associated conditions, especially in patients with immunodeficiency, diabetes, or complex forms of mucosal hyperplasia, were emphasized. The probability of developing adaptive changes in the fungus in case of incomplete destruction of colonies was revealed. Synthesis of data on phenotypic and genotypic changes in *Candida* during repeated laser irradiation confirms the need for individual selection of therapy regimens. The analysis showed that the Er:YAG laser effectively reduces the viability of *C. albicans*, including thick biofilms, but for *C. glabrata* or *C. tropicalis*, it is often necessary to increase the power and duration of the procedure. In addition, laser disinfection helps to improve the course of chronic forms of glossitis and hyperplastic lesions, making this method promising for the long-term prevention of recurrent infections and improving the quality of life of patients. Comparison of the effectiveness of different energy delivery systems and evaluation of microbiological parameters allowed us to propose optimal exposure parameters, while emphasizing the importance of preliminary susceptibility testing of isolates. However, given the predominance of in vitro studies in the current evidence base, further in vivo investigations and well-designed clinical trials are critically needed. In particular, studies comparing the Er:YAG laser with other laser modalities (e.g., Er,Cr:YSGG, CO_2_) or evaluating its use in combination with chemical or photodynamic therapies will help clarify its clinical utility and define standardized protocols. It is recommended to implement standardized Er:YAG laser disinfection protocols in dental practice, combining them with chemical agents and controlling the exposure parameters depending on the *Candida* species. Prospects for further research include the study of innovative combinations of laser therapy with biosensor diagnostic methods and coatings for implant surfaces. A limitation was the lack of large-scale clinical trials, which makes it difficult to develop uniform protocols for all forms of candidiasis.

## Figures and Tables

**Table 1:** Key parameters and performance indicators of Er:YAG and Er,Cr:YSGG lasers in antifungal studies.

Parameter	Er:YAG	Er,Cr:YSGG	Main Results
Wavelength (nm)	About 2940	About 2780	Both lasers operate in the mid-infrared range, which facilitates the ablation of fluid and biofilm
Type of study (in vitro/in vivo)	Used in biofilm research and clinical applications	Used mainly in experiments on dental and prosthetic surfaces	Both types of lasers are capable of reducing the number of viable *Candida* cells under the right parameters
Optimum power (W)	1.5–5.0 (depending on the pulse mode)	1.0–4.0 (adjustable for different modes)	Efficiency increases with increasing power, but at the same time, the risk of thermal damage to tissue increases
Exposure time (s)	10–30	5–20	Longer exposures usually provide better disinfection, but require control over the preservation of surrounding tissue
Penetration depth (mm)	Several millimeters, mostly surface action	Similar or slightly smaller	Limited depth of penetration is relevant for surface biofilms, which allows targeted destruction of the fungal layer without significant damage to the underlying layers

Source: created by the author based on [23,41].

**Table 2:** Specificity of oral candidiasis (albicans and non-albicans) and the potential of laser therapy.

Candida form	Typical Lesions	The Main Factors of Virulence	Probable Sensitivity to Laser
*C. albicans*	Denture stomatitis, chronic atrophic candidiasis	High enzymatic activity, adhesion to mucous membranes	Considered to be fairly high with correct Er:YAG and Er,Cr:YSGG parameters
*C. glabrata*	Recurrent gum disease, possible drug-resistant forms	Weaker hyphal formation, but higher tolerance to some antimycotics	May require higher laser parameters for effective suppression
*C. tropicalis*	Ulcerative lesions of the tongue, erythematous lesions	Expressed ability to form biofilms	Potentially high sensitivity; in thick biofilms, careful longer exposures are required
*C. parapsilosis*	Frequent infections in immunocompromised patients	Active formation of biofilms on dentures	Needs to be investigated for optimal parameters; preliminary data indicate good potential
*C. krusei*	Rare but resistant gingival infections	Low adhesion, but high tolerance to some drugs	Limited information; requires tailored approaches and additional research

Source: created by the author based on [8,54,55].

**Table 3:** Summary of Laser Protocols.

Study	Laser Type	Chemical Adjuncts	Microorganisms Targeted	Key Findings
Deeb et al. [1]	Er:YAG	NaOCl, CHX, H_2_O_2_	Bacteria	Significant microbial reduction with laser + antiseptics
Reddy et al. [2]	Er:YAG, CO_2_	NaOCl, CHX, H_2_O_2_, NaF	Bacteria	Combined treatments more effective than individual
Datla et al. [3]	Er,Cr:YSGG		*C. albicans*, *S. aureus*	Laser outperformed ultrasonic and syringe rinsing
Golge et al. [4]	Er:YAG-PIPS, Er,Cr:YSGG	NaOCl	*C. albicans*	Er:YAG-PIPS + NaOCl most effective; Er,Cr:YSGG less effective
Valenti et al. [5]	Er:YAG	None	*C. albicans*, *Streptococcus* spp., *Lactobacillus* spp.	Laser less invasive, effective against *Candida*

**Table 4:** Comparison of Er:YAG And Er,Cr:YSGG Lasers.

Laser Type	Wavelength (nm)	Typical Power Range (W)	Typical Exposure Time (s)	Target Applications	Advantages
Er:YAG	2940	1.5–5.0	10–30	Deep fungal disinfection, biofilm matrix breakdown	High antifungal effect, effective in dense biofilms
Er,Cr:YSGG	2780	1.0–4.0	5–20	Tissue-sensitive applications, prosthetic surfaces	Lower thermal impact, better postoperative comfort

## Abbreviations

CFU—Colony Forming Unit, CHX—Chlorhexidine, CLSM—Confocal Laser Scanning Microscopy, EDTA—Ethylenediaminetetraacetic Acid, Er:YAG—Erbium-Doped Yttrium Aluminum Garnet, Er,Cr:YSGG—Erbium, Chromium-Doped Yttrium, Scandium, Gallium and Garnet, HSP—Heat Shock Protein, MALDI-TOF MS—Matrix-Assisted Laser Desorption Ionization Time-of-Flight Mass Spectrometry, NaOCl—Sodium Hypochlorite, PI—Propidium Iodide, PRISMA—Preferred Reporting Items for Systematic Reviews and Meta-Analyses, aPDT—Antimicrobial Photodynamic Therapy, UW-QOL—University of Washington Quality of Life.
